# Health Needs Assessment in Home-Living Older Adults: Protocol for a Pre-Post Study

**DOI:** 10.2196/55192

**Published:** 2024-04-18

**Authors:** Fifi Kvalsvik, Bente Hamre Larsen, Grethe Eilertsen, Helle K Falkenberg, Ingvild Dalen, Stine Haaland, Marianne Storm

**Affiliations:** 1 Department of Public Health Faculty of Health Sciences University of Stavanger Stavanger Norway; 2 Research Group of Older Peoples’ Health University of South-Eastern Norway Drammen Norway; 3 Department of Nursing and Health Sciences University of South-Eastern Norway Drammen Norway; 4 National Centre for Optics, Vision and Eye Care Department of Optometry, Radiography and Lighting Design, Faculty of Health and Social Sciences University of South-Eastern Norway Kongsberg Norway; 5 Section of Biostatistics Research Department Stavanger University Hospital Stavanger Norway; 6 Department of Quality and Health Technology Faculty of Health Sciences University of Stavanger Stavanger Norway; 7 Department of Health and Welfare services Stavanger kommune Stavanger Norway; 8 Faculty of Health Sciences and Social Care Molde University College Molde Norway; 9 Research Group of Nursing and Health Sciences Research Department Stavanger University Hospital Stavanger Norway

**Keywords:** assessment, frailty, healthy aging, health care, home-living older adults, pre-post study, protocol

## Abstract

**Background:**

Conducting a health needs assessment for older adults is important, particularly for early detection and management of frailty. Such assessments can help to improve health outcomes, maintain overall well-being, and support older adults in retaining their independence as they age at home.

**Objective:**

In this study, a systematic approach to health needs assessment is adopted in order to reflect real-world practices in municipal health care and capture the nuances of frailty. The aim is to assess changes in frailty levels in home-living older adults over 5 months and to examine the observable functional changes from a prestudy baseline (t1) to a poststudy period (t2). Additionally, the study explores the feasibility of conducting the health needs assessment from the perspective of home-living older adults and their informal caregivers.

**Methods:**

Interprofessional teams of registered nurses, physiotherapists, and occupational therapists will conduct 2 health needs assessments covering physical, cognitive, psychological, social, and behavioral domains. The study includes 40 home-living older adults of 75 years of age or older, who have applied for municipal health and care services in Norway. A quantitative approach will be applied to assess changes in frailty levels in home-living older adults over 5 months. In addition, we will examine the observable functional changes from t1 to t2 and how these changes correlate to frailty levels. Following this, a qualitative approach will be used to examine the perspectives of participants and their informal caregivers regarding the health needs assessment and its feasibility. The final sample size for the qualitative phase will be determined based on the participant’s willingness to be interviewed. The quantitative data consist of descriptive statistics, simple tests, and present plots and correlation coefficients. For the qualitative analysis, we will apply thematic analysis.

**Results:**

The initial baseline assessments were completed in July 2023, and the second health needs assessments are ongoing. We expect the results to be available for analysis in the spring of 2024.

**Conclusions:**

This study has potential benefits for not only older adults and their informal caregivers but also health care professionals. Moreover, it can be used to inform future studies focused on health needs assessments of this specific demographic group. The study also provides meaningful insights for local policy makers, with potential future implications at the national level.

**Trial Registration:**

ClinicalTrials.gov NCT05837728; https://clinicaltrials.gov/study/NCT05837728

**International Registered Report Identifier (IRRID):**

DERR1-10.2196/55192

## Introduction

Across the globe, the population is aging rapidly. There are around 1.4 billion people aged 60 years and older in the world today, and this figure will double by 2050 [1]. Norway is no exception to this demographic transition. It is anticipated that the number of people aged 65 years and older will exceed the population aged 0-19 years by 2030 [2]. As is widely known, the older segment of the population tends to have a higher prevalence of chronic diseases and disabilities [3,4]. This implies that the demographic shift could come at a great cost to older adults themselves and society [3,5]. Direct challenges at the societal level may include a strain on public finances and the welfare system, increased demand for health services, and rising health care costs [6,7].

One way to mitigate the impacts of an aging population is to support aging in place [8,9]. The term “aging in place” refers to meeting an older adult’s needs and supporting them to live independently or with some assistance in their own home for as long as possible [8]. Most older adults prefer to live at home because it enables them to maintain their independence, autonomy, and identity, as well as their connection to social support [10]. In addition to being the preferred choice for many older adults, aging in place is viewed as a cost-effective strategy for addressing the aging population’s needs, as it can reduce the expenses associated with institutional care [11]. Given these positive effects, encouraging older adults to continue living at home could be an essential part of the solution to meeting the challenges of population aging.

Many older adults are able to live independently without much support; others, however, may have age-related issues that prevent them from aging in place [12]. These age-related issues often lead to frailty, resulting in increased functional limitations, increased risk of falls, disability, institutionalization, hospitalization, and mortality [13,14]. As frailty progresses, efforts to mitigate, manage, or reverse this decline become increasingly difficult to implement [15]. Hence, there is a broad consensus on the importance of assessing frailty [14,16,17], particularly in relation to older adults aged 70 years and older [16,18]. In general, early assessment of frailty can prevent, reduce onset, or slow its progression in older adults [19], contributing to better quality of life and healthy aging [18,20].

There is currently no consensus on the definition of frailty [17,21]. At its simplest, frailty is a condition that indicates increased vulnerability to negative health outcomes [22]. Despite varying definitions, there is general consensus that frailty is multidimensional and involves complex interactions between physical, psychological, social, cognitive, and behavioral factors [17,19]. The diversity of definitions and the wide range of contributing factors also explain why there is a variety of measures of frailty [16,17].

Incorporating frailty into health needs assessments is, therefore, crucial, as this can help identify specific areas of need, set clear goals to meet those needs, and ultimately contribute to the health promotion of this population [23]. The term “health needs assessment” refers to a systematic method of identifying unmet health and care needs of a population, leading to agreed priorities and resource allocation that will improve health outcomes [24].

While our knowledge of frailty has improved [14], our understanding of the factors that contribute to frailty is still incomplete, and the understanding of how these factors relate to frailty is still limited [19]. These knowledge gaps are a clear indication that considerable efforts are still needed in this area.

Given the complexity of frailty as a construct and its enormous impact on the lives of older adults, adopting a collaborative approach is deemed essential to improving our understanding and achieving meaningful progress. This is further supported by research that highlights the benefits of fostering collaboration between health care professionals, individual service users, families, researchers, and other stakeholders to improve health service outcomes [25-27].

Such collaboration enables all relevant aspects of older adults’ health to be assessed, and essential information can be collected to develop effective, locally applicable, and relevant solutions [28]. With this in mind, we have established partnerships with health care professionals and other stakeholders in a Norwegian municipality, actively involving them in the planning and implementation phase of the project.

Interprofessional teams consisting of registered nurses, physiotherapists, and occupational therapists will be involved in this study. They will perform 2 health needs assessments of home-living older adults, initially at baseline and then after a period of 5 months. These assessments cover several factors, including physical (mobility and sensory), cognitive, psychological (depression), social (loneliness), and behavioral (alcohol consumption). The objectives of our study are 3-fold: first, to assess changes in frailty levels in home-living older adults over 5 months; second, to explore the observable functional changes from a prestudy baseline (t1) to a poststudy period (t2) and how these changes correlate to frailty levels; and finally, to gain deeper insight into the perspectives of older adults and their informal caregivers to enhance the feasibility of implementing this type of health needs assessment within municipal health and care services.

The research questions that guide the study are (1) What observable changes, if any, can be identified in the frailty levels of home-living older adults over a period of 5 months? (2) Are there identifiable changes in functions that can be observed from t1 to t2, and how these changes relate to frailty levels? and (3) What are the perceptions of home-living older adults regarding the feasibility of the health needs assessment, with regard to its content, delivery, and procedures?

## Methods

### Study Design

This pre-post study, registered on ClinicalTrials.gov (NCT05837728), is based on a case-only design to conduct health needs assessments, initially at the baseline and then after 5 months. The study is conducted as part of the research project “More good days at home: Advancing health-promoting practices in municipal health care services for older recipients of home care,” funded by the Norwegian Research Council (320622).

To gain in-depth insights, we use a 2-step research process. In the first step, a quantitative approach will be applied to examine changes in frailty levels in home-living older adults over 5 months. In addition, we will examine observable functional changes from t1 to t2 and how these changes correlate to frailty levels.

Following this, a qualitative approach will be used to investigate the perspectives of participants and their informal caregivers concerning the health needs assessment and its acceptability.

### Setting

This study is placed in the context of the Norwegian health care system, known for its universal health care. This system ensures everyone has equal access to health care regardless of socioeconomic status or geographical location [29]. The system is based on a clearly defined division of responsibilities between the local municipalities and the central government.

The local municipalities are tasked with organizing primary, preventive, and nursing care services [29] and are committed to providing the necessary health services, such as home and personal care, free of charge to all home-living older adults [30]. These services are universally available and tailored to the specific needs of each older adult. To access these services, older adults are required to submit an application to the district office in their municipality [31]. Once the application is submitted, it will be reviewed by health care professionals in the health and welfare services office. After this phase, the health care professionals will reach out to the older adults to proceed further.

While municipalities manage the above care services, central government oversees specialist care, which includes hospital services. This clear division ensures comprehensive health care for all citizens [29]. This study takes place in a municipality in Western Norway with a population of approximately 146,011. In this population, 9874 individuals are older adults aged 75 years or older [32].

### Study Sample

#### Inclusion Criteria

To be eligible for the study, the older adults must be aged 75 years or older and have applied for municipal health and care services. These services may include medical emergency alarms, senior activity centers, home-based nursing services, and practical assistance with daily chores. In addition, older adults who received follow-up care after a hospital stay, were discharged from rehabilitation, or received emergency medical care are also eligible.

#### Exclusion Criteria

This study does not include home-living older adults who are younger than 75 years of age, have a cognitive impairment, or are undergoing palliative care. In this study, cognitive impairment refers to the inability to understand the study and provide informed consent to participate.

### Recruitment

Participant recruitment was conducted in collaboration with 3 health and welfare service departments in a municipality in the western region of Norway between February 1 and June 26, 2023. Health care professionals purposively selected participants according to the eligibility criteria. During their initial phone contact with potential participants, health care professionals used their professional judgment to assess the individual’s ability to understand the research project and provide informed consent to participate.

Subsequently, health care professionals asked a designated research team member, a nurse with experience in mental health, to obtain informed consent from those who meet the initial criteria. To ensure consistency in the process, the same nurse visited all potential participants for the consent process. Potential participants were then given time to consider this information before giving their written consent.

The design of our health needs assessment package includes a cognitive assessment. This approach ensures that participants identified with cognitive impairment in the first assessment are not included in the second assessment.

We aim to enroll 40 participants in the quantitative phase of this study, in line with the agreement made with the municipality at the beginning of the project, taking into account their resource limitations. In the next phase, which uses a qualitative approach, we plan to invite all 40 participants for an interview, with the actual number of interviews conducted depending on the participants’ consent to participate. To capture diverse perspectives, we will strive to include a group of home-living older adults who differ in terms of age, gender, marital status, living situations, and health care needs.

The aforementioned sample size takes into account the limited resources available for the project, including budget constraints, personnel resources, and time limitations, while ensuring the collection of meaningful data to address the research questions.

### Data Collection

To respond to the diverse needs of older adults, we have interprofessional teams consisting of registered nurses, physiotherapists, or occupational therapists to carry out the health needs assessments. The assessment is conducted twice at the participant’s home. To facilitate successful implementation, we provided simulation-based training and debriefings. We have also produced webinars available in Norwegian to enhance learning. The link to the webinars can be provided upon request. Our study is ongoing, so we will publish it upon completion.

The interprofessional teams will use an iPad (Apple Inc) to administer assessments covering the various factors—physical (mobility and sensory), cognitive, psychological (depression), social (loneliness), and behavioral (alcohol consumption). The collected data will be registered electronically and directly submitted to Services for Sensitive Data (*Tjenester for Sensitive Data* [TSD]) for secure storage and processing.

After the initial assessment, interprofessional teams will collaboratively review the results to determine whether, and to what extent, the older adult is eligible for assistance and to determine what type of support is available and appropriate for the individual [33]. Examples of such support include practical help with everyday tasks, provision of medical emergency alarms, access to walking aids, recommendations for physiotherapy sessions, referrals to hearing or vision centers, encouragement to participate in senior activity centers, and facilitating contact with primary care physicians.

After 5 months, the same interprofessional teams will conduct the second health needs assessment to determine whether the support provided at the first assessment is suitable for helping participants maintain their quality of life.

With regard to the qualitative approach, we will use 2 data collection methods in order to minimize mono-method bias [34]—in-depth individual interviews and dyadic interviews. We will use a general interview guide approach to collecting data from all the participants, which will give us the flexibility to modify or adapt questions based on responses to previous questions [35]. To maintain consistency, the topics and questions in the 2 interview methods will be identical and guided by the Theoretical Framework of Acceptability (TFA) proposed by Sekhon et al [36]. The questions will include, for example, (1) how the participant thinks and feels about the assessment, (2) what the participant thinks about the effort required to participate in this type of assessment, (3) the participant’s understanding of the assessment process, (4) what benefits or value they believe the assessment will have for them, and (5) whether they feel the assessment was conducted respectfully or not. Finally, the interviews will be audio recorded and transcribed verbatim to ensure accuracy in the data analysis.

### Measure Descriptions

The selection of measures was informed by a comprehensive review of existing literature, consultation with experts, and input from stakeholders involved in the project. Many of the measures have been validated in different contexts, including the Norwegian context. The study also includes novel scales that will be tested for validity within this particular sample of home-living older adults. The measures in the study are described below, and an overview is given in Table 1.

**Table 1:** Overview of the measures used in the study.

Variables			Questionnaires and tests used in assessments
**Demographics**			
	Gender	Male, female, or other?	
	Age	How old are you (years)?	
	Household	Do you live alone or with others?	
	Children	Do you have any children?	
	Residential suitability	Is your residence suitable for your needs?	
	Transportation	Do you use private or public transport?	
**Physical function**			
	Mobility: lower extremity function	To assess the lower extremity function, we will use the SPPB^a^, a well-established instrument commonly used in home-living older adults [37-40]. The Norwegian version of SPPB, translated by Bergh et al [41], has been tested for reliability, and the result shows that it has high reliability when used by trained physiotherapists on older populations [42]. Other reasons for using SPPB are that it is fast, safe, and easy to administer; requires little training; and uses simple equipment [43]. The test assesses standing balance, walking, and rising from a chair. The result from the test is quantified by scores. In addition, the test is claimed to be highly sensitive to changes over time [37,44].	
	Sensory: hearing	To detect hearing impairment, we will use the KAS^b^-Screen interview guide [45]. The questions used in our study stem from the hearing and verbal communication or social life subscales. Previous evaluation and validation found this test to be an adequate tool for detecting hearing impairment in older adults and provides important information on how this impairment can affect the daily life of older adults [45,46].	
	Sensory: vision	To detect vision impairment, we will use a structured vision assessment tool called KROSS^c^ developed by the University of South-Eastern Norway in collaboration with the Vestre Viken Hospital Trust and stroke survivor organizations. The KROSS tool includes assessments of visual acuity, visual field, eye alignment and movements, and visual in attention [47,48].	
**Cognitive function**			
	Cognitive function	To identify possible cognitive impairment, the Mini-Cog test will be used. This takes approximately 3 minutes to administer and consists of 2 cognitive tasks: 3-item word recall and a clock drawing test [49]. Mini-Cog was preferred to other cognitive screening tools because it is simple and concise, requires no special equipment, is easily incorporated into general practice and different senior settings, and has high sensitivity and specificity [49,50]. Additionally, it has been suggested that Mini-Cog performs well as a screening test among older adults in communities [51]. The Norwegian version of Mini-Cog was translated by Rostoft et al [52].	
**Psychological function**			
	Depression	To assess depression in old age, we will use the 4-item GDS^d^. Although other versions of GDS can be used to identify common symptoms of depression in older individuals, the 4-item GDS is reported to be most suitable for detecting depression in older adults who live at home [53,54]. Overall, the 4-item GDS has been shown to be a valid and reliable tool for evaluating depression in older adults, with a sensitivity of 100% and 62% specificity [54]. Another benefit of using this scale is that it is a relatively short scale, which reduces the burden on the respondent and the time it takes for health professionals to administer the test [54].	
**Social function**			
	Loneliness	The validated UCLA^e^ 3-Item Loneliness Scale will be used to assess loneliness. This scale is designed to measure loneliness based on individual perceptions of how often a person feels (1) a lack of companionship, (2) left out, and (3) isolated from others [55]. In terms of reliability, this scale has been found to have good internal consistency, and test-retest reliability has shown that the scale produces consistent results over time [56,57]. In terms of validity, it was demonstrated that this scale correlates well with other measures of loneliness and discriminates well between loneliness and other constructs [55]. Additionally, this measure is chosen because it is quick to administer, has been used in large surveys, has been tested in older populations, and measures overall loneliness quite well [55,58]. The Norwegian version of this scale was translated by Arumugam et al [59].	
**Behavioral function**			
	Alcohol consumption	For a brief assessment of alcohol consumption, we will use the AUDIT^f^ developed by the World Health Organization [60]. Our study will use AUDIT-4, which consists of 3 questions from AUDIT-C and the 10th item of the original scale [61]. In terms of reliability and validity, previous research with different samples has shown that AUDIT-4 has good internal consistency, criterion validity, and convergent validity [62,63].	
Assessment of frailty			To assess frailty, we will use the CFS^g^. The CFS, a tool based on clinical judgment, assesses key areas such as multimorbidity, cognition, and other functional areas [64]. CFS has been shown to be a strong predictor of several outcomes, including falls, length of hospitalization, multimorbidity, and mortality [65]. The CFS was translated into Norwegian by Rostoft et al [66] and has been used to assess frailty in various settings in Norway, including emergencies and intensive care [67,68]. Its simplicity and predictive power in assessing physical health and potential outcomes for frail older adults [17,69] make it an appropriate choice for use in municipal health care settings.

^a^SPPB: Short Physical Performance Battery.

^b^KAS: *Kombinert Alvorlig Sansesvikt* (Combined Serious Sensory Impairment).

^c^KROSS: *Kompetanse om Rehabilitering Om Syn og Slag* (Competence, Rehabilitation of Sight after Stroke).

^d^GDS: Geriatric Depression Scale.

^e^UCLA: University of California, Los Angeles.

^f^AUDIT: Alcohol Use Disorders Identification Test.

^g^CFS: Clinical Frailty Scale.

### Outcome Measures

This study has two primary outcome measures: (1) identifying changes in frailty levels of home-living older adults over a period of 5 months and (2) assessing any observed changes in functions from t1 to t2 and how these changes relate to the level of frailty.

In addition to the primary outcome measures, this study will also evaluate the feasibility of the health needs assessment. Emphasis will be placed on the acceptability of the assessment among older adults and their informal caregivers, as well as the practicality of implementation in relation to time and resource constraints.

### Data Analysis

#### Quantitative Analyses

The statistical package SPSS (version 29; IBM Corp) will be used for all statistical analyses. Descriptive statistics will be used to provide an overview of the demographic variables. Given the exploratory nature of this study and the limited sample size, our analysis involves looking at the data from multiple perspectives. The aim is to uncover patterns and relationships that could inform the development of hypotheses for future studies.

To answer the research questions, we will first calculate descriptive statistics for changes in frailty levels between 2 time points, using mean and SD or median and IQR for a skewed distribution. This will be followed by paired comparisons using either 2-tailed *t* test or the Wilcoxon test. We will also examine the association between the changes in frailty and baseline levels by plotting and estimating correlation coefficients (Pearson and Spearman). We will further assess changes in functional variables and the bivariate associations between these changes and the change in frailty.

#### Qualitative Analyses

The interview transcripts will be read thoroughly and reread to ensure familiarity with the content. NVivo (version 12; Lumivero) software will be used to help organize and code the data. The data analysis will be carried out using a thematic analytical approach based on the guidelines of Braun and Clarke [70].

### Patient and Public Involvement

In this study, older adult participants were not involved in developing the study design or objectives. The study design and objectives were developed by the researchers of this study in collaboration with the local municipality stakeholders. The Service User Association for Older Adults in Norway (*Pensjonistforbundet*) contributed insights to the grant application, which was successfully funded by the Research Council of Norway. Furthermore, details about the study design and recruitment procedures were provided to health care professionals prior to data collection.

### Ethical Considerations

Ethics approval was obtained from the Regional Committees for Medicine and Health Research Ethics (REK) of West Norway on November 15, 2022 (523455). The project was subsequently approved by the Norwegian Agency for Shared Services in Education and Research (SIKT) on January 19, 2023 (100847), which ensures that adequate safeguards are in place to protect the privacy of participants and to maintain the confidentiality of data. This study will be conducted in accordance with the Declaration of Helsinki guidelines [71] from REK and the Data Protection Impact Assessment (DPIA). It will also be conducted under the principle of informed consent. Prior to data collection, all the participants who agreed to participate in the study will sign a consent form, which includes a description of the study, its objectives, and participants’ involvement and rights. Participation in the study is entirely voluntary and participants can withdraw at any time. Additionally, participants are entitled to access the information documented about them and to make necessary corrections to any errors discovered.

### Data Management

#### Overview

The project manager will supervise the day-to-day operation of the project and be responsible for ensuring that DPIA guidelines are followed.

#### Data Collected From the Frailty Assessment

The collected data will be registered electronically and directly submitted to TSD for secure storage and processing. The code list that links personal identification information to the individual participant will be secured by the project manager to ensure there is no unauthorized access to the information. Only the selected project team members will have access to the anonymized data sets. No other party will have access to the research data prior to dissemination. Furthermore, other paper-based research data will be anonymized and kept in locked cabinets or archives that are only accessible to project team members.

#### Data Collected From the Interview

Only a designated member of the research project and the project manager will have access to the audio files stored at Nettskjema. The interview will be transcribed into written text and all personal identifiers will be removed to ensure anonymity. The transcription will be shared with other project team members as appropriate. This data will be retained until December 31, 2026, and then deleted from TSD.

## Results

The initial baseline assessments were completed in July 2023, and the second health needs assessments are ongoing. We expect the results to be available for analysis in the spring of 2024.

## Discussion

### Principal Considerations

Studies have documented that frail older adults have an elevated risk of negative health outcomes [13,14]. Early assessment of frailty in older adults is, therefore, crucial, and is recommended. Despite its recognized importance, assessing frailty is not considered a standard clinical practice in many health care systems [18], including in Norway [72]. This situation is understandable as such an assessment often requires significant resources, which many health care facilities lack [73].

Our study is carefully designed to reflect real-world applications and settings, with a particular focus on municipal health care in Norway, where health care professionals tend to have heavy workloads. These conditions pose a challenge to incorporating additional tasks, such as a frailty assessment, into routine health needs assessments. As mentioned earlier, older adults typically turn to their local municipality for home care services and health care professionals make home visits to assess their care needs. This assessment process uses the standardized tool (The Norwegian Information System for the Nursing and Care Sector [*Individbasert pleie- og omsorgsstatistikk*; IPLOS]), which was developed to optimize care needs assessments in Norway [33]. Despite being a standardized tool, IPLOS has been criticized for being too subjective, as assessments appear to vary widely among health care professionals [74].

In response to these challenges, we aim to refine the health needs assessment process to improve objectivity and consistency, and to highlight the critical needs to incorporate frailty assessments into routine municipal care practices. The path begins with selecting appropriate measurement tools that are meaningful and practical, and training interprofessional teams to implement them. Part of this training process includes the task of conducting assessments and discussing the results to make informed decisions.

Beyond integrating assessments, our study aims to shed light on the dynamic nature of frailty and provide a deeper understanding of the factors that impact its progression. This insight could potentially help older adults and their informal caregivers to manage frailty. Additionally, it can better prepare health care professionals to develop and implement strategies aimed at delaying and mitigating the progression of frailty.

### Strengths and Limitations

This study has several strengths. First, it deepens our understanding of frailty as a complex condition and highlights how levels of frailty and related functional changes can differ. Moreover, because the study is designed to reflect the real-world settings and applications, it can help improve health care practices in the municipal sector. The involvement of local stakeholders in the municipality from the planning to the implementation phase ensures that the study remains relevant and applicable, thereby facilitating the translation of knowledge into practice. Additionally, the inclusion of home-living older adults’ and their informal caregivers’ perspectives can help facilitate improvement in the feasibility of the assessment.

As with other studies, this study has its limitations, particularly with regard to the relatively small sample size and the absence of a control group. This could lead to misinterpretation of the observed changes, either in the support or services for participants, other confounding factors, or due to a habituation effect over the 5-month period. Consequently, this limitation may also potentially impact the internal validity of the study, as well as the construct validity of the study [75]. To address this challenge, we will carefully compare our findings with those of previous studies investigating similar approaches to aiding the interpretation of outcomes.

Despite its inherent limitations, this study will serve as an important first step in developing a more systematic health needs assessment for home-living older adults and the development of health promotion strategies that can improve these older adults’ quality of life.

Nonetheless, the results should be interpreted with caution due to the absence of a control group, the limited sample size, and the potential underrepresentation of all home-living older adults. These limitations suggest that the results indicate possible correlations and patterns, rather than confirming a definite relationship or establishing specific cause-and-effect relationships.

## Abbreviations

DPIA: Data Protection Impact Assessment
IPLOS: The Norwegian Information System for the Nursing and Care Sector (Individbasert pleie- og omsorgsstatistikk)
REK: Regional Committees for Medicine and Health Research Ethics
SIKT: Shared Services in Education and Research
TFA: Theoretical Framework of Acceptability
TSD: Services for Sensitive Data (Tjenester for Sensitive Data)

## Data Availability

The data sets generated and analyzed during this study are not publicly available due to ethical considerations inherent in this research but are available from the corresponding author on reasonable request.
